# Upstream open reading frames may contain hundreds of novel human exons

**DOI:** 10.1371/journal.pcbi.1012543

**Published:** 2024-11-20

**Authors:** Hyun Joo Ji, Steven L. Salzberg

**Affiliations:** 1 Center for Computational Biology, Johns Hopkins University; Baltimore, Maryland, United States of America; 2 Department of Computer Science, Johns Hopkins University; Baltimore, Maryland, United States of America; 3 Department of Biomedical Engineering, Johns Hopkins University; Baltimore, Maryland, United States of America; 4 Department of Biostatistics, Johns Hopkins University; Baltimore, Maryland, United States of America; AUTh: Aristoteleio Panepistemio Thessalonikes, GREECE

## Abstract

Several recent studies have presented evidence that the human gene catalogue should be expanded to include thousands of short open reading frames (ORFs) appearing upstream or downstream of existing protein-coding genes, each of which might create an additional bicistronic transcript in humans. Here we explore an alternative hypothesis that would explain the translational and evolutionary evidence for these upstream ORFs without the need to create novel genes or bicistronic transcripts. We examined 2,199 upstream ORFs that have been proposed as high-quality candidates for novel genes, to determine if they could instead represent protein-coding exons that can be added to existing genes. We checked for the conservation of these ORFs in four recently sequenced, high-quality human genomes, and found a large majority (87.8%) to be conserved in all four as expected. We then looked for splicing evidence that would connect each upstream ORF to the downstream protein-coding gene at the same locus, thus creating a novel splicing variant using the upstream ORF as its first exon. These protein coding exon candidates were further evaluated using protein structure predictions of the protein sequences that included the proposed new exons. We determined that 541 out of 2,199 upstream ORFs have strong evidence that they can form protein coding exons that are part of an existing gene, and that the resulting protein is predicted to have similar or better structural quality than the currently annotated isoform.

## Author summary

We analyzed over 2000 human sequences that have been proposed to represent novel protein-coding genes, and that reside just upstream of known genes. These “upstream ORFs” (uORFs) would represent a surprisingly large addition to the human gene catalogue, which after decades of refinement now contains just under 20,000 protein-coding genes. They would also create over 2000 new bicistronic genes, which number only 10 in current human annotation databases. We hypothesized that rather than novel genes, these sequences might instead represent novel exons that can be spliced into existing protein-coding genes, creating new isoforms of those genes. Using a combination of transcriptional evidence and computational predictions, we show that at least 541 of the previously-described uORFs can be used to create novel protein-coding exons, generating new transcripts and new protein isoforms, but not requiring the addition of entirely new genes to the human gene catalogue. We also demonstrate that the predicted three-dimensional structure of some of the new protein isoforms hints at new or improved functions for existing proteins.

## Introduction

Although the human protein-coding gene count has been converging on just under 20,000 genes in recent years [1,2], multiple recent studies have suggested the possible presence of thousands of additional short protein-coding genes [3–10]. Most of these proposed novel genes take the form of short open reading frames (ORFs) that occur just upstream or downstream of existing protein-coding genes, apparently on the same messenger RNA. While some of these studies have suggested that the unannotated ORFs encode biologically active proteins [5–8,10,11], others suggest that the ORFs might instead perform regulatory roles in modulating RNA or protein levels [9,12].

One problem with the hypothesis that uORFs represent distinct proteins is that each would create a bicistronic transcript; i.e., a single transcript that encodes two distinct proteins. These are not unknown in the human genome, but they are very rare: as of now, only 10 bicistronic genomic loci have been annotated in the MANE (v1.3) annotation [13], the current “gold standard” for human protein-coding gene annotation. From the thousands of uORFs described in a recent survey, only 25 have thus far been added to the GENCODE annotation database [3].

We therefore wished to examine a hypothesis that might explain many of the uORFs, but that would not require the addition of large numbers of novel genes and bicistronic transcripts to the human gene catalogue: what if these upstream ORFs represent protein-coding exons that have not previously been annotated? In this case we would need to find evidence for splice sites that would link together each uORF with the known, downstream ORF at the same locus.

Previous work [3] identified a set of uORFs that have at least two lines of evidence suggesting their protein-coding nature: (1) mutations within the uORFs that show a bias towards synonymous mutations; and (2) ribosome profiling data, in the form of Ribo-seq experiments [14], that suggest that the uORFs are being translated. Worth noting here is that Ribo-seq evidence cannot be used as direct evidence to prove the existence of a protein-coding sequence. A decrease in ribosome footprint density is typically interpreted as indicating a stop codon, but if a hypothesized uORF is part of an exon rather than a distinct gene, then a decrease might instead indicate a splice donor site; i.e., the end of the exon.

To search for novel exons that would explain uORFs, we considered several lines of evidence. First we confirmed that the novel exons were conserved in multiple distinct human genomes that have recently been sequenced. Second, we looked for direct evidence of splicing from the large-scale expression data generated by the GTEx project [15], and for computational evidence of splice donor sites within the uORFs that could be paired with previously-annotated acceptor sites. Either of these findings could link a uORF to a downstream protein-coding gene and create a novel isoform of that gene. Third, we evaluated the novel protein sequences created by each hypothesized new isoform, and we considered whether the folded protein structure, as predicted by ColabFold [16], would be comparable to the structure of the canonical protein at that locus.

## Methods

We began our analysis with a large set of potentially novel human protein-coding regions collated from seven publications identifying ORFs from a variety of evidence sources, including Ribo-seq experiments and evolutionary conservation [4–10]. These ORFs occur in regions previously thought to be untranslated, and in most cases appearing within the 5’ untranslated regions (UTRs) of protein-coding transcripts. A meta-analysis of these publications [3] collected a total of 7,264 of these ORFs, including some contained entirely within known protein-coding regions and some that were in the 3’ UTR. 3,771 of the 7,264 ORFs were either entirely upstream or else partly upstream and partly overlapping with the known protein-coding region. For ease of discussion, we will refer to all of these as uORFs. We focused our analysis on a high-confidence subset of 2,199 uORFs that were reported by at least two publications.

### Preservation of uORFs in other human genomes

All uORFs considered here were originally identified on the reference human genome GRCh38. Because multiple additional human genomes have now been sequenced to high levels of completeness and accuracy, we reasoned that any novel protein-coding gene should be preserved in those other genomes as well. We evaluated the level of conservation by aligning the sequences from GRCh38 to each target genome, and then checking for any differences in the target genome. We also required that each uORF sequence be found in the 5’ UTR region of a transcript in the same gene locus on the target genome.

We aligned the uORFs to four different human genomes: a Puerto Rican individual (PR1, v3.0) [17], a Southern Han Chinese individual (Han1, v1.2) [18], an Ashkenazi individual (Ash1, v2.2) [19], and the complete, gap-free CHM13 human genome (v2.0) [20]. We selected these genomes because they are a diverse set representing distinct human populations, and because they are among the few that have been both assembled and annotated, allowing for comparison of their gene content. The genomes were annotated using Liftoff [21] to map genes from GRCh38 onto each assembly.

**Fig 1:**
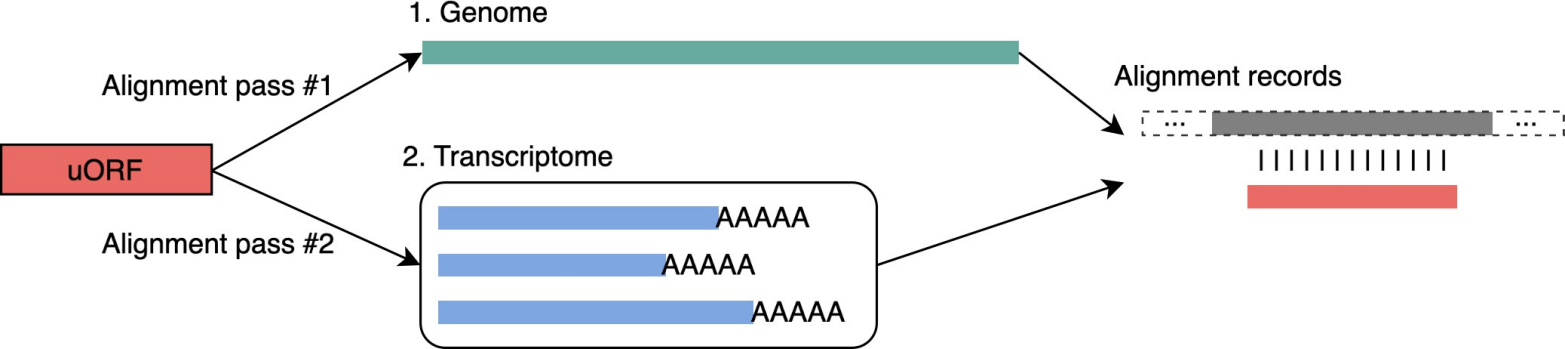
Each upstream ORF (uORF) was aligned to multiple human genomes, using both the genomic sequence and the annotated transcripts. The transcriptome alignment handled cases where a uORF spanned two exons in the 5’UTR of an annotated transcript.

We aligned each ORF sequence twice: first to the genome and then to the transcriptome (Fig 1). For both alignments, we used minimap2 (v2.26) [22] in its single-end short read (sr) mode. Each alignment result was then used to assign a conservation level on a scale from 0 to 7. If the genomic alignment and transcriptomic alignment results did not agree, the ORF with the maximum conservation level was used. Transcriptomic alignments helped to identify ORFs that spanned splice junctions, which could occur when the 5’ UTR region of a transcript contained more than one non-protein coding exon. See S1A Text for the specific parameters used for alignments.

As expected, most query ORFs yielded perfect nucleotide-level matches to the target genomes. For those that did not, we computed all translations and checked if any encoded a protein identical to the one encoded by the original query ORF. For each genome, there were handful of cases (35 on average) in which a query uORF had a perfect protein-level match without a sequence-level match. The resulting alignment coordinates were then annotated using the target genomes’ annotations. Genomic and transcriptomic alignments were processed slightly differently. For a genomic alignment, any overlapping gene and transcript features were collected. When an annotation was found, we checked to confirm that the uORF occurred in the 5’ UTR region of the annotated transcript and that the name of the downstream gene corresponded to the one described in the original source [3].

To quantify conservation levels, we used three separate criteria with different weights, and the sum of the weights was used as a proxy for the level of conservation for uORFs (Table 1). All cases in which uORFs were assigned a score of 6, 4, 2, or 0 (i.e., lacking a gene locus match) were manually examined to ensure that they were not located in different genomic locations from what was annotated by [3] (S1C Text). Any ORF scoring at least 4 was considered conserved, unless the sequence match occurred more than 1kbp away from the source gene. Using these criteria, 1931 out of the set of 2199 uORFs (87.8%) were conserved in all four target genomes (S5 Table).

**Table 1:** uORF conservation criteria and their weights. Each uORF was assigned a score between 0 and 7 and uORFs scoring > 3 were considered conserved in a genome.

Criterion	Weight
Sequence match	4
5’ UTR containment	2
Gene locus match	1

### De novo construction of transcripts

**Fig 2:**
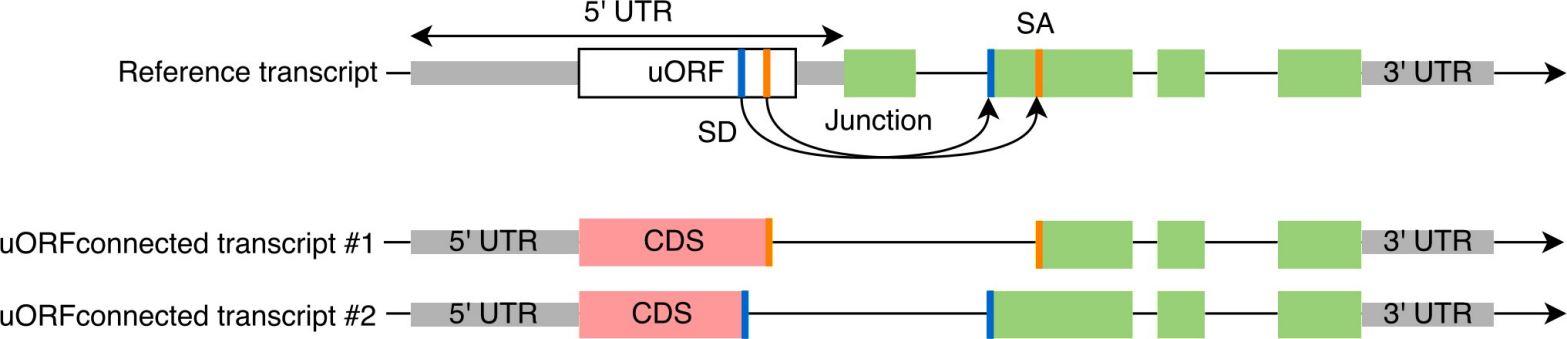
Construction of uORF-connected transcripts from a uORF and a downstream protein-coding transcript. The original protein-coding sequence is shown in green rectangles. For uORF-connected transcript #1, a splice junction (red bars) found in the GTEx collection of RNA-seq data is used to link the uORF to the second exon of the downstream transcript. For uORF-connected transcript #2, a splice donor (SD) site predicted by Splam (blue bars) is paired with an annotated splice acceptor (SA) site in a MANE transcript. The novel protein sequences are shown in red rectangles.

All 2199 uORFs were evaluated for possible splicing evidence, even those excluded in the previous filtering step. Using either experimentally observed splice sites from GTex or predicted splice sites from the Splam program [23], we constructed putative novel coding sequences by concatenating the CDS from the uORF (up to the splice site) to the coding exons of the downstream gene, creating a novel *uORF-connected transcript* (**Fig 2**). Each uORF-connected transcript was associated with a reference transcript from the GENCODE (v39) annotation and with the protein sequence annotated for that transcript.

### Incorporation of experimental splicing evidence and predicted splice junctions

We collected our experimentally identified splice junctions from a large set of assembled transcripts created as part of the initial construction of the CHESS human annotation [2], which is based on assemblies of 9,814 RNA-seq samples collected by the GTEx project [15]. Note that most of these splice sites do not appear in the final CHESS catalogue. We merged and summarized the assembled transcripts using TieBrush [24], which also captured the read coverage for each junction. We also filtered out all non-canonical splice junctions, requiring that donor sites begin with either GT or GC and that acceptor sites end with AG.

If a splice junction’s donor site occurred within a uORF and its acceptor site was contained in the downstream ORF at the same gene locus, we then attempted to connect the upstream and downstream ORFs to yield a novel ORF (**Fig 2**). We discarded any construct if (1) it contained a premature stop codon, (2) its length was not a multiple of three, or (3) it encoded a CDS whose length was < 90% of the length of the reference transcript. This filtering ensured that each putative novel isoform would produce a valid protein sequence of similar length to the reference protein.

We then considered additional splice sites (i.e., those not seen in GTEx) if they were given high scores by Splam, a highly accurate splice site predictor [23]. We created these potential splice sites by first scanning the uORF regions to detect potential splice donors (GT dinucleotides), and then pairing them with splice acceptors coming from annotated MANE transcripts. Each junction was then scored by Splam, and we retained those with an average score for the donor and acceptor of at least 0.9. We then applied the same filtering steps as described above, removing ORFs if they would not generate a protein of similar length to the reference.

**Fig 3:**
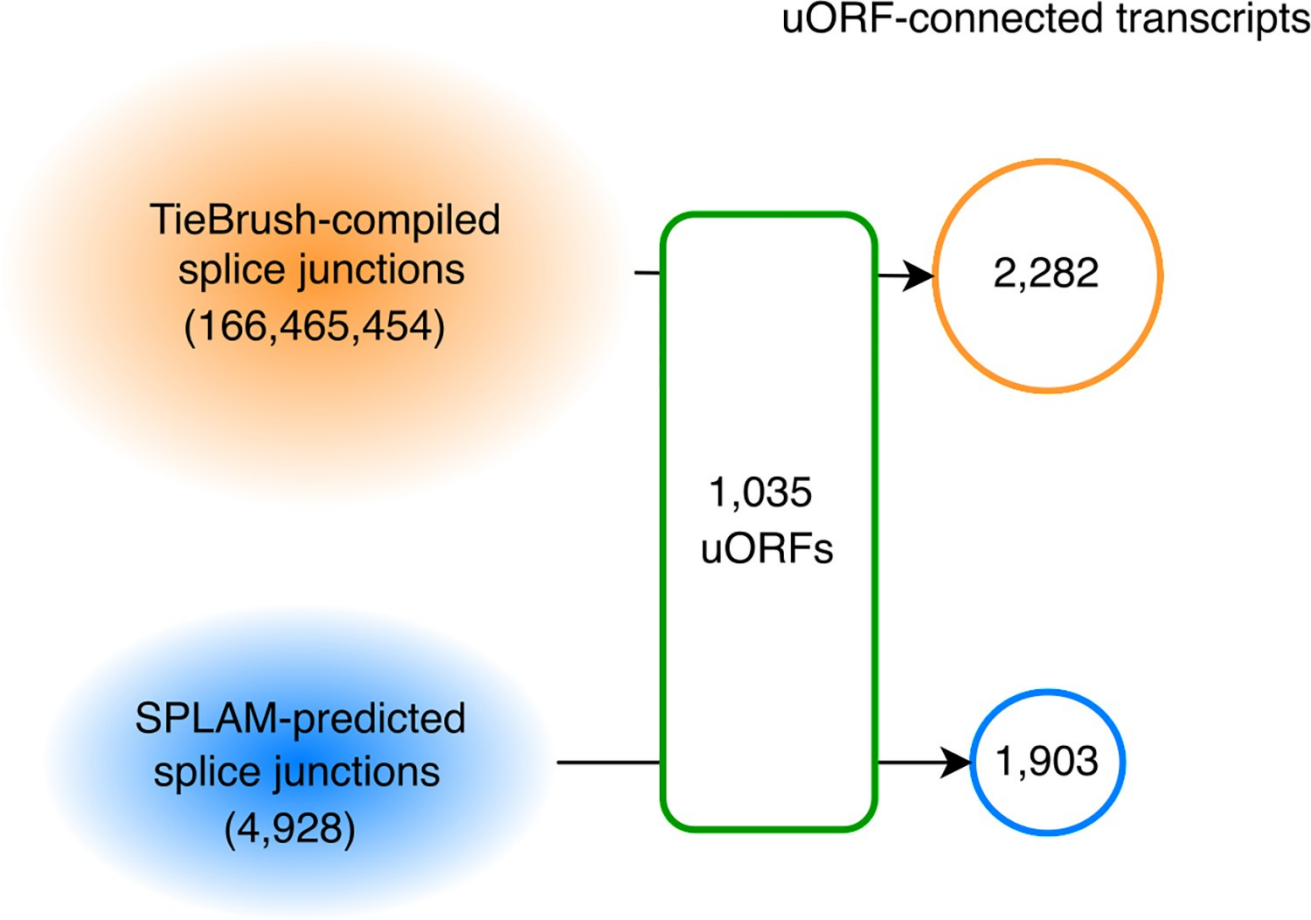
Novel isoforms were constructed using direct and predicted splicing evidence. Splice junctions seen in ~10,000 GTEx RNA-seq and Splam predictions yielded a total of 4,185 uORF-connected transcripts from 1,035 uORFs. 2,282 were supported by GTEx data and 1,903 had Splam support but not GTEx evidence.

There were total of 166,465,454 junctions in the GTEx-supported set and 4,928 additional junctions in the Splam-predicted set. We created 2,282 candidate transcripts using TieBrush junctions and an additional 1,903 using the Splam-predicted splice junctions (**Fig 3**). In total, we constructed 4,185 uORF-connected transcripts from 1,035 uORFs, which are provided in S2 Table.

In a handful of cases (24), uORF-connected transcripts encoded proteins with a non-AUG start codon, or more specifically, isoleucine (I) instead of methionine (M). These transcripts were included in our analysis but are separately annotated, in S7 Table. We also noted that a very small number of novel transcripts (2) encoded proteins with exactly the same amino acid sequence as their references (due to duplications between the uORF and the downstream ORF), and these are separately shown in S8 Table.

### Folding transcripts

We used ColabFold, a tool that accelerates AlphaFold2’s protein structure predictions [16,25], to predict the structure of all novel proteins encoded by uORF-connected transcripts. We used the pLDDT score, AlphaFold2’s per-residue confidence metric, as a proxy for the quality of each folded structure. As described in the AlphaFold2 publication [25], a protein with pLDDT >70 is considered to have high confidence and stability. See S1B Text for the specific parameters used for folding proteins using ColabFold.

If a uORF-connected transcript encoded a protein with a similar pLDDT score to the one encoded by its reference, then we considered it more likely that the novel protein was a valid functional isoform. In contrast, if the novel protein had a lower pLDDT score, then we eliminated it from further consideration (although some of these may nonetheless be valid). More precisely, a uORF-connected transcript that encoded a protein that achieved either an increase (>1) or no change (±1) in average pLDDT was retained for further investigation. Occasionally, a uORF-connected transcript was compared to multiple reference proteins when the locus contained multiple alternative proteins. In that case, we required that the uORF-connected transcript have a similar or better pLDDT than at least one of the references.

## Results

### Most uORFs are conserved in other human genomes

As described in Methods, we checked all uORFs to determine if they were conserved in four different human genomes: PR1, Han1, Ash1, and CHM13. Because humans are very closely related, we only considered a uORF to be conserved in another human if it had a complete sequence-level match; i.e., full-length and 100% identical. Additional criteria such as the uORF’s containment in the 5’ UTR region or consistency of the gene locus was used to further classify uORFs into different levels of conservation (Table 2 and Methods).

**Table 2:** Number of uORFs in GRCh38 that were conserved in each of four other human genomes, at varying levels of conservation. Level 7 indicates a perfect match contained in the 5’ UTR region of a protein-coding transcript at the correct gene locus. Values in parentheses are the number of cases in which the best sequence match occurred at a different genomic location (>1kbp away), not the one previously annotated.

Conservation score		Han1	CHM13	Ash1	PR1
**Conserved**	7	1,931	1,943	2,078	2,065
	6	14	15	1	2
	5	66	68	6	4
	4	25 (5)	27 (5)	5 (0)	2 (0)
	Subtotal	2,036 (5)	2,053 (5)	2,090	2,073
**Not conserved**	3	146	131	108	125
	2	2	1	0	0
	1	4	5	0	0
	0	6	4	1	1
	Subtotal	158	141	109	126

**Fig 4:**
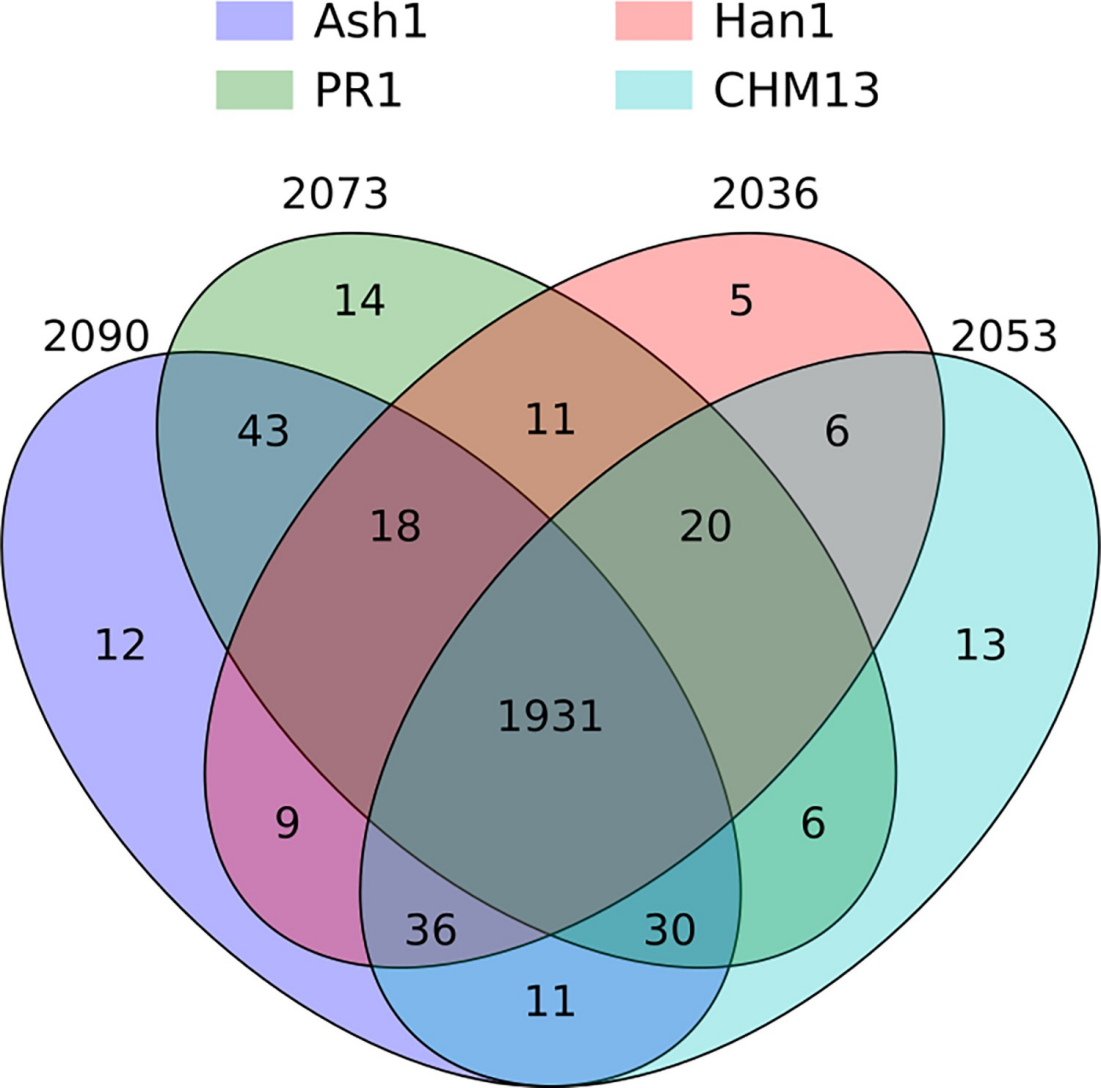
Conserved uORFs shared between GRCh38 and all subsets of four different genomes. The innermost region shows that there were 1,931 uORFs conserved in all five genomes.

A large majority of the uORFs, 1,931 out of 2,199 (87.8%), were conserved in all four individual human genomes as well as GRCh38 (Fig 4). Most of the remaining uORFs (213/268) were conserved in at least two of the four genomes, with only a handful conserved in exactly one genome. Interestingly, there were 43 ORFs shared only between Ash1 and PR1, 36 conserved in all genomes except PR1, and 30 conserved in all genomes except Han1. The conservation levels for each of the 2,199 uORFs can be found in S5 Table. Only uORFs from the 1,931 fully-conserved set were included in our final list of uORF-connected transcripts.

### Some uORFs form novel protein coding exons

Our main hypothesis is that uORFs identified within known protein-coding transcripts might instead be explained as novel protein-coding exons that represent isoforms of the existing gene, which *a priori* seemed more likely than an entirely new protein-coding gene sharing the same transcript. As described in Methods, we constructed novel exons using splice junctions supported by either experimental (RNA-seq) or computational evidence, and retained transcripts only if they encoded a protein at least 90% as long as the reference.

Out of 2,282 proteins encoded by uORF-connected transcripts with GTEx support, 782 had a pLDDT score that was the same or higher than their reference proteins. The RNA-seq read coverage on the novel junctions in these 782 uORF-connected transcripts was higher on average (~249) than the mean coverage for all junctions used to construct uORF-connected transcripts (~139). These transcripts corresponded to 294 distinct uORFs.

Although many fewer uORF-connected transcripts (1,903) were constructed using Splam-predicted junctions, 757 of these encoded proteins with an equal or higher pLDDT score than their references. These Splam-supported transcripts corresponded to 314 distinct uORFs, of which 67 were also used to construct GTEx-supported transcripts. Splam was used to score all junctions not included in the splice junction set extracted from GTEx samples, so this overlap represents cases where a Splam-supported junction and a GTEx-supported junction link the same uORF to its downstream protein. The union of these two sets contained 541 unique uORFs from the set of 1,931 uORFs conserved in all five genomes (S1 Table).

In total, we constructed 4,185 uORF-connected transcripts encoding distinct proteins with either GTEx or Splam support. 1,539 of these encoded proteins with a pLDDT score that was the same or higher than their references, and we further investigated 462 of the 1,539 instances with strictly higher average pLDDT scores. Although some of our constructs achieved higher pLDDT scores than the corresponding reference proteins, they should not be construed as being “better” than existing isoforms. The higher pLDDT scores functions instead as evidence that the novel exon produces a well-folded protein that is likely to be as good as the original. Both the new construct and the reference protein might be valid isoforms.

The structures for these 462 proteins were manually compared their references and categorized into six groups: end truncation, alpha helix elongation, alpha helix deletion / truncation, straightening, tightening, and structural addition. Some proteins displayed more than one of these changes. The most common type of change was end truncation (236), followed by alpha helix deletion / truncation (205), structure addition (55), alpha helix elongation (34), alpha helix straightening (9), and tightening (6). For cases of alpha helix deletion / truncation that resulted in an increase in average pLDDT, we observed that the structure deletions were often accompanied by removal of long, unstructured coils, as illustrated for the OPA1 gene locus in S1B Fig.

**Fig 5:**
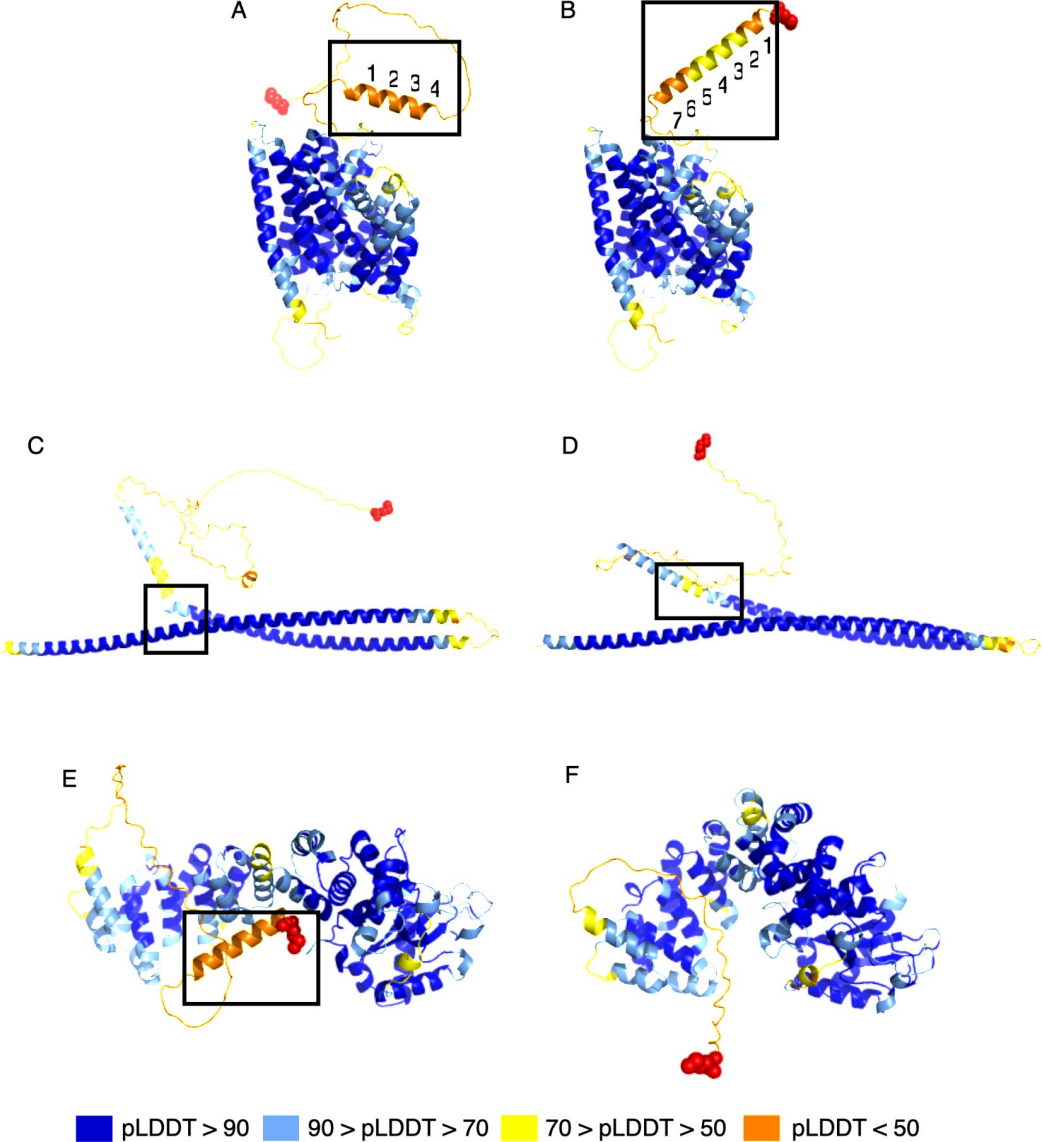
Examples of structure changes in novel protein variants identified in this study. (A) and (B): alpha helix elongation at the SLC28A1 gene locus, where (A) shows the reference protein, ENST00000398637.10, and (B) shows the novel isoform, uorft_2119. The average pLDDT increase from A to B was 2.96. (C) and (D): straightening at the TRAK2 gene locus, where (C) shows the native protein, ENST00000430254.1, and (D) shows the novel isoform, uorft_441. The average pLDDT increase from C to D was 3.41. (E) and (F): tightening of a structure of TBRG4, where (E) shows the known protein, ENST00000395655.8, and (F) shows the novel isoform, uorft_1435. The main structural changes are highlighted by black boxes for each pair of structures. Red spheres represent the N-terminus of each protein. The average pLDDT increase from E to F was 4.29.

Fig 5 illustrates several of the structural differences we observed. In Fig 5A and 5B, an alpha helix was lengthened in a uORF-connected isoform of SLC28A1, which led to an increase of 2.96 in the pLDDT score. In Fig 5C and 5D, we illustrate a "straightening" event, where two alpha helix structures in the native isoform of TRAK2 were interrupted by a kink and a short coil region. In the uORF-connected isoform, these were connected and formed a single helix, which yielded an increase of 3.41 in pLDDT. Fig 5E and 5F illustrates a cases in which the structural components of a protein were brought closer together, or "tightened." Tightening is particularly interesting for proteins with binding domains, as it might yield an increased binding efficacy for a protein to its target. The ORF-connected transcript at the TBRG4 gene locus shown in Fig 5F encodes a protein predicted to have a tighter conformation, leading to an increase of 4.29 in average pLDDT. The TBRG4 protein, also known as FASTKD4, contains an RNA-binding domain (RAP), whose efficacy may be influenced by how tightly the protein is packed [26,27].

The most common type of change we observed was end truncation, where an unstructured (coil) region at the N-terminus of a protein was removed, resulting in a higher average pLDDT score. For instance, the isoform defined by a uORF-connected transcript at the ZDHHC5 gene locus has a much shorter coil compared to the canonical protein at that locus, leading to an increase of 4.5 in average pLDDT score (S1A Fig).

Finally, structure addition refers to cases in which one or more alpha helices or beta sheets were added to the reference protein. Adding well-folded structures to a protein generally leads to an increase in its pLDDT score. For example, a uORF-connected transcript at the ZNF32 gene locus adds a series of four beta sheets near the N-terminus of the reference protein, yielding an increase of 3.28 in average pLDDT (S1C Fig).

## Discussion

We evaluated 2,199 upstream ORFs that were previously identified using multiple lines of evidence, all of them on existing protein-coding transcripts. If these uORFs turn out to be genuinely novel protein-coding genes, they will also create bicistronic transcripts, representing a huge increase in the number of such transcripts in the human genome, which currently number only 10. Our analysis suggests that at least 541 of these uORFs (24.6% of the total) could instead be used to form novel protein-coding exons that can be connected to exons from existing genes to form new protein variants. These new isoforms can be added to the human genome annotation without creating entirely new genes or bicistronic transcripts.

Our process incorporated multiple lines of evidence including RNA sequencing support, inter-individual sequence conservation, computational splice site predictions, and protein structure prediction. The inital set of uORFs was generated based on at least two other sources of evidence: Ribo-seq data supporting the translation of the uORFs, and evolutionary conservation suggesting that the regions were protein-coding [3]. Both of these lines of evidence also support our proposed novel transcripts, which use the same start codons as the uORFs and only shorten the uORF slightly with the adoption of a splice donor site before the stop codon.

We should emphasize that our process was conservative, and other uORFs from the set of 2,199 might also represent protein-coding exons. For example, we required that the predicted structure of each novel isoform have either the same or higher average pLDDT than the reference, but isoforms with a lower pLDDT might nonetheless be valid. Further research is necessary to determine if these additional uORFs might also be merged with existing genes to create transcripts with a novel first exon.

Finally, we note that all of the lines of evidence used here and in previous studies of uORFs are indirect and subject to limitations. The splicing evidence used to link novel upstream exons to existing transcripts included evidence from a computational splice site predictor, Splam, which although accurate is imperfect, and from a large RNA sequencing database, GTEx, which despite its size does not cover every human tissue and condition. Further studies are needed to confirm the findings reported here.

## Supporting information

S1 Fig Additional examples of structural changes in novel protein variants identified in this study. (A): Coil truncation at the ZDHHC5 gene locus. The reference protein (ENST00000323578.13) is on the left and the novel isoform (uorft_2063) is on the right. The pLDDT increase was 4.5. (B): Alpha helix deletion at the OPA1 gene locus. The reference protein (ENST00000361510.8) is on the left and the novel isoform (uorft_760) is on the right. The pLDDT increase was 3.23. (C): Alpha helix was replaced by beta sheets at the ZNF32 gene locus. The reference protein (ENST00000374433.7) is on the left and the novel isoform (uorft_1781) is on the right. The pLDDT increase was 3.28. The main structural changes are highlighted by black boxes for each pair of structures. Red spheres represent the N-terminus of each protein.
(TIFF)

S1 Text Supplementary methods for the conservation analysis of uORFs and protein structure predictions. (A): uORF sequence alignment with minimap2. (B): Protein structure prediction with ColabFold. (C): Manual inspection of uORF mapping positions on human genomes.
(DOCX)

S1 Table Novel protein-coding exons. This table lists uORFs containing novel protein-coding exons that led to approximately the same (-1 < **δ** < 1) or higher (**δ** > 1) average pLDDT scores compared to their GENCODE references. Each novel protein-coding exon can be traced back to > = 1 uORF-connected transcripts. For each uORF, we selected one uORF-connected transcript (column B) that achieves the highest **δ** (column D) compared to its reference (column C). Column E provides the complete list of (uORF-connected, reference) pairs derived from a uORF.
(XLSX)

S2 Table uORF-connected transcripts. This table contains uORF-connected transcripts (IDs in column H) constructed using either GTEx or Splam-supported splice junctions. For each construct, we made available which uORF it originates from (column C) and the splice junction coordinates (column F) connecting the uORF to a downstream CDS that belongs to a reference transcript in GENCODE (column J). Note that the junction coordinates follow a 1-based, fully closed interval system used in a GTF/GFF format. The level of support for each junction, read coverage if GTEx-supported and predicted score if Splam-supported, is available in column G. The table also includes the ColabFold-predicted average pLDDT scores for each construct and its reference, as well as their differences– **δ**. A transcript or reference with a missing average pLDDT value (notated with a ‘.’) are the ones skipped due to their CDS length being > 2k amino acids. Columns M and N tell if the reference is an NMD (according to GENCODE) or a MANE transcript. Columns R and S contain the novel CDS lengths and protein sequences encoded by each uORF-connected transcript.
(XLSX)

S3 Table Select uORF-connected transcripts with an equal or similar average pLDDT score compared to their GENCODE references. This table presents a subset of uORF-connected transcripts that achieved approximately the same (-1 < **δ** < 1) or higher (**δ** > 1) average pLDDT scores compared to their references. For those with a higher average pLDDT, we visualized them and assigned each instance to category(s). Category 0 indicates undefined (i.e., can’t be determined), and categories 1 ~ 6 represent N-terminus end truncation, alpha helix deletion/truncation, alpha helix elongation, straightening, tightening, and structural addition in that order. A construct can be tagged with more than 1 category.
(XLSX)

S4 Table uORF conservation scores in each of the four target human genomes. [3] annotated a few uORFs on multi-gene loci (column C), each of which we confirmed if those genes are overlapping (results shown in columns D and E). For uORFs assigned with conservation levels of 0, 4, or 6, we show their alignment positions (aln_coords) and the coordinates for their associated genes (gene_coords), which were recorded by [3]. The dist columns show the distance between the expected gene locus for a uORF and its alignment on a target genome.
(XLSX)

S5 Table Number of conserved uORFs in each of the four target human genomes. This table shows the number of uORFs in each conservation level ranging from 0 to 7. Columns C, E, G, and I show the curated statistics, where uORFs in levels 0, 4, or 6 were bumped up if their alignment positions and gene locus coordinates were less than 100 bp away.
(XLSX)

S6 Table Gene names missing in each of the four target human genomes’ annotations. This table lists the gene names that were missing in the target genomes’ annotations while being present in the uORF annotation produced by [3]. Asterisks (*) indicate that the gene is located in a multi-gene locus (i.e., overlapping gene start/end positions) or a synonym exists.
(XLSX)

S7 Table uORF-connected transcripts encoding proteins with a non-AUG start codon. (XLSX)

S8 Table uORF-connected transcripts encoding the same protein sequences as their GENCODE references. (XLSX)
