# Supplementary figures and images for: Upstream open reading frames may contain hundreds of novel human exons
Source: PLoS Comput Biol. 2024 Nov 20;20(11):e1012543. doi: 10.1371/journal.pcbi.1012543 (PMC11578521; doi:10.1371/journal.pcbi.1012543)

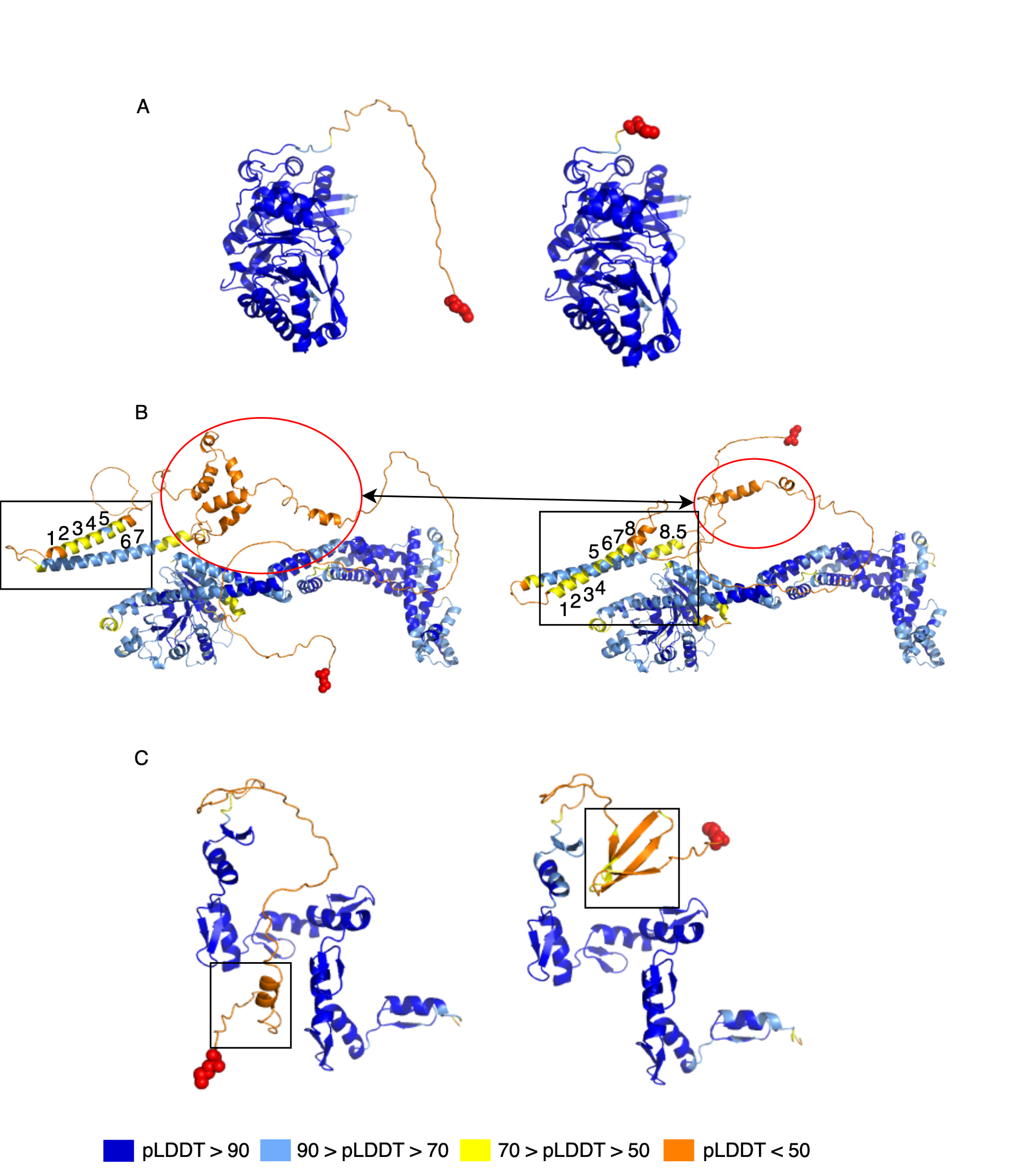

Supplement: S1 Fig — (A): Coil truncation at the ZDHHC5 gene locus. The reference protein (ENST00000323578.13) is on the left and the novel isoform (uorft_2063) is on the right. The pLDDT increase was 4.5. (B): Alpha helix deletion at the OPA1 gene locus. The reference protein (ENST00000361510.8) is on the left and the novel isoform (uorft_760) is on the right. The pLDDT increase was 3.23. (C): Alpha helix was replaced by beta sheets at the ZNF32 gene locus. The reference protein (ENST00000374433.7) is on the left and the novel isoform (uorft_1781) is on the right. The pLDDT increase was 3.28. The main structural changes are highlighted by black boxes for each pair of structures. Red spheres represent the N-terminus of each protein. (TIFF) [file pcbi.1012543.s001.tiff]
